# Comparing Patient to Provider Perceptions of Birth Trauma: What are We Missing?

**DOI:** 10.1007/s00737-026-01763-6

**Published:** 2026-09-17

**Authors:** Michelle L Miller, Kylie L Willams, Tiffany R Williams, Cara Berg Raunick, Sarah A Greenwell, Maggie Sullivan, Russell J Ledet, Camila Arnaudo, Kundai Crites, Julian J Spangler, Alissa Conklin

**Affiliations:** 1 Department of Psychiatry, Indiana University School of Medicine, Indianapolis, United States; 2 Department of Obstetrics & Gynecology, Indiana University School of Medicine, Indianapolis, United States; 3 Midwest Trauma-Informed Training, Health Care Education & Training, Indianapolis, United States; 4 Department of Research, Indiana University School of Medicine, Indianapolis, United States

**Keywords:** Birth Trauma, Postpartum, Birth Satisfaction, Provider Communication

## Abstract

**Background:**

Traumatic birth experiences are common and negatively affect the health and wellbeing of the birthing person. Interactions between patients and providers are an important risk factor for experiencing birth as traumatic. The current study investigated both patient and provider perceptions of birth.

**Methods:**

Participants (*N* = 146) were recruited from a mother-baby postpartum recovery unit and asked about birth satisfaction, if they found their birth traumatic (using DSM-5 Diagnostic Criteria for PTSD), and if their providers debriefed with them post-birth. Providers (*N* = 95, including obstetricians and midwives) were also asked if the birth was traumatic.

**Results:**

There were 60 participants (41.1%) that experienced birth trauma, yet only two thirds received a debrief from their providers (*n* = 40, 66.7%). Only 65.9% of providers accurately endorsed their patient’s birth as traumatic. Participants who endorsed birth trauma reported significantly lower satisfaction with their birth experience than those who did not (mean difference = 2.881, *t* = 7.217 (144), *p*<.001, CI [2.092, 3.670]). Most participants endorsed that they wanted to be asked whether their birth experience was traumatic, especially those that had experienced birth trauma (81.8%).

**Conclusion:**

There is a mismatch between how patients and providers are viewing the same birth experience. Obstetric providers are working with patients when they may be recovering from a traumatic event, creating an ideal opportunity for trauma-informed care. A universal workflow should include briefly discussing birth experiences, especially if the experience was traumatic, with future research focusing on patient and provider outcomes associated with improved conversations.

## Highlights

Traumatic birth experiences are extremely common.

Interactions with providers can strongly shape a patient’s delivery experience.

Patients and providers were asked if the patient experienced birth trauma and if there was a conversation post-delivery.

Many providers are not accurately perceiving or talking about birth trauma.

Most postpartum individuals want to be asked if their birth experience was traumatic, demonstrating an opportunity for improved care.

## Introduction

A traumatic birth experience occurs when childbirth directly causes short and/or long-term distressing reactions that then have negative impacts on the health and wellbeing of the birthing person (Leinweber et al. 2022). Traumatic birth experiences are quite common, as 1 in 3 women (and up to 45%) perceive their birth as traumatic (Ayers et al. 2024; Beck et al. 2018). Adverse experiences associated with birth trauma include strong feelings of failure, self-blame, loss, and anger; increased risk of postpartum depression and suicidality; strained romantic relationships; and not wanting to or being able to have as many children as desired (Ayers et al. 2007; Beck and Watson 2008; Dekel et al. 2019; Di Blasio et al. 2017).

Feelings of helplessness and powerlessness during interactions with providers are a powerful predictor of birth trauma as the relationship between obstetric providers and patients is integral to a patient’s well-being pre-, during-, and post-delivery. In a seminal study of over 2000 women who had experienced a traumatic birth, nearly half of participants believed that their trauma could have been reduced or prevented if they had better communication with their provider (Hollander et al., 2017). Further, discrepancy of expectations around birth and lack of involvement in the decision-making process contributed more to the experience of birth trauma than fear for one’s own health or life, indicating a potential intervention target. Importantly, exposure to traumatic childbirth events (e.g., shoulder dystocia, infant death) can also have a significant professional and personal effect on maternity care clinicians (e.g., attending obstetrician physicians, registered nurses; Robinson et al., 2023).

In this study, we measured our participants’ *and their obstetric providers’* perception of the birth experience immediately post-delivery to better understand (1) how often providers and patients perceived the same experience as traumatic; (2) communication patterns after the experience of birth trauma; and (3) qualitatively examine how participants felt being asked about birth trauma immediately postpartum.

## Methods

### Procedures

Participants were recruited by research coordinators from the Riley Maternity Tower Postpartum Recovery Unit, a large academic medical center that is also one of only two Level IV hospitals for labor and deliveries in the state. Inclusion criteria: (a) participant was available in the postpartum unit following delivery, (b) at least 18 years old, and (c) spoke and read English. Recruitment occurred from June 2024 to July 2025. Participants were included in the study regardless of birth complications, number of days spent on the mother-baby unit, and method of delivery. There were 230 potential participants that were approached to participate in the study. Of those, 22 (9.5%) declined to participate, 62 potential participants were missed, (e.g., asleep, in the NICU, asked to come back and then discharged before could return), and 146 participants enrolled in the study (see Table 1). To better understand clinic size, in 2024, there were 1,443 deliveries from patients that attended the clinics where recruitment took place.

**Table 1:** Missingness on Surveys

Reason Survey Was Unable to Be Obtained	*N* (%)
Could not locate patient	15 (17.9)
Patient declined survey	22 (26.2)
Other	47 (56.0)
Timing issue (discharged, delivered on weekend, etc.)	16 (34.0)
Patient in the NICU	10 (21.3)
Attending to maternal needs (sleeping, showering, etc.)	12 (25.5)
Attending to newborn needs (breastfeeding, soothing, etc.)	3 (6.4)
No visitors	5 (10.6)
Technology issue	1 (2.1)

Patients who expressed interest in the study were consented in person and then immediately completed an online survey verbally conducted by research coordinators on an iPad with quantitative questions on demographics, birth satisfaction, birth trauma assessment, and qualitative assessments on hospital care. Participants were also offered resources for managing birth trauma, including a self-guided prompt for processing their birth story through writing and information for an outpatient psychotherapy clinic that specialized in treating perinatal mental health. Participants were contacted 4–6 weeks after childbirth to provide feedback on the acceptability of being asked about birth trauma post-delivery.

To measure provider perceptions of birth trauma, the obstetrician or midwife who delivered the infant was asked to complete a survey immediately post-delivery on whether they perceived their patient’s birth as traumatic and if they discussed it with the patient. Research coordinators identified the delivering physician or midwife and sent the survey to the appropriate provider. Data was collected through REDCap, a secure, web-based application designed for research provided by the Institute for Clinical and Translational Science (Harris et al. 2009, 2019).

## Measures

###  Demographics

Participants were asked to report on their age, race, ethnicity, gender, and parity. Providers were asked to report their age, gender, occupation type, years of experience, and if they received trauma informed care training.

###  Birth Satisfaction Scale-Revised Indicator (BSS-RI)

The BSS-RI is a well-validated 6-item measure of birth satisfaction utilizing items from the original Birth Satisfaction Scale-Revised (BSS-R; Martin & Martin, 2014; Martin et al. 2017). It is intended to be short, easily administered, and able to evaluate stress as well as perinatal service delivery. The measure is composed of two subscales, *stress and emotional response to labor and birth* and *quality of care*. Participants can respond “Agree”, “Somewhat agree”, or “Disagree”. Higher scores represent greater birth satisfaction (item range 0–2). Cronbach’s alpha for this sample was good (α = 0.72).

###  DSM-5 PTSD Criteria 

We utilized the questions from the Diagnostic and Statistical Manual of Mental Disorders (DSM-5; APA, 2013) to assess if an event can be considered a traumatic event, which is part of the criteria necessary to receive a diagnosis of PTSD. The questions were: (1) *Do you think your most recent birth was traumatic?*; (2) *Did you think that you were close to being seriously injured or dying?*; (3) *Did you fear for your life?*; and (4) *Did you fear for the life of your infant?*. A birth experience was considered traumatic if the participant endorsed ≥ 1 item on this questionnaire; scores ranged from 0 to 4. Participants and providers were asked the same questions except providers were asked to respond to prompts about the patient’s experience. After these questions, participants and providers were each asked if a debrief was conducted; providers were asked if a debrief happened if they endorsed at least one of these questions.

###  Acceptability Measure

A 10-item study-specific measure was used to understand the acceptability and reaction to asking about birth trauma in the immediate postpartum period. This measure was added in April 2025 and completed by participants between 2 weeks and 10 months postpartum. Sample items included “*Did you want to be asked if your birth experience was distressing or traumatic?”* and *“What did you find most helpful to be asked about?”.* Responses were either “Yes/No” or free text.

### Statistical Analyses

Statistical analyses were performed using IBM SPSS version 29. Data was normally distributed and counts and percentages were included for all variables. An independent samples t-test was used to compare variables between groups. A chi-square test of independence was used to examine the congruence between endorsement of birth trauma by participants and endorsement of birth trauma by providers. Thematic analysis was used to analyze the participants’ responses to the qualitative follow-up questions (Braun & Clark, 2006; Nowell et al. 2017). The transcript data was systematically and iteratively reviewed, culminating in 42 initial codes. The codes were then reduced to primary themes and discussed further along with example quotes. There was minimal missingness due to research assistants directly collecting data from participants. No a priori sample size was calculated as there were no prior reports in this population to guide calculations.

## Results

### Participant Demographics

Participants (*N* = 146; see Table 2) were primarily female (*n* = 145, 1 participant identified as Non-Binary), non-Latina (*n* = 136, 93.2%) and multiparous (had given birth before) (*n* = 87, 59.6%) There were 95 (65.1%) provider responses.

**Table 2:** Participant Demographics

Variables	*N* (%)
**Female**	145 (99.3)
**Age (years)**	29.22 (5.89, 18–46)
**Race**	
White	87 (59.6)
Black	41 (28.1)
Asian/Pacific Islander	2 (1.4)
Middle Eastern or North African	1 (0.7)
Other	9 (6.2)
**Ethnicity (% Hispanic)**	10 (6.8)
**First Delivery**	59 (40.4)

Overall, participants endorsed a high amount of satisfaction with their birth experience (*M =* 8.51, *SD* = 2.76, range = 1–12), including across the stress and emotional response to labor and birth subscale (*M =* 4.72, *SD* = 2.48, range = 0–8) as well as quality of care (*M =* 3.79, *SD* = 0.66, range = 0–4). This is consistent with prior average scores of birth satisfaction (total, *M =* 8.24, *SD* = 2.86; quality of care, *M =* 3.48, *SD* = 0.97; Martin et al. 2017).

However, when comparing between participants who experienced birth trauma compared to those who had not experienced birth trauma, participants who endorsed birth trauma endorsed a significantly lower amount of satisfaction on average with their birth experience (*M =* 6.82, *SD* = 2.76, range = 1–12) compared to those who did not endorse experiencing birth trauma (*M =* 9.70, *SD* = 2.06, range = 3–12; mean difference = 2.881, *t* = 7.217 (144), *p*<.001, CI [2.092, 3.670]). Additionally, participants who did not experience birth trauma were more likely to say they received a debrief from their providers (*n* = 62, 72.1%) than participants who did experience birth trauma (*n* = 40, 66.7%).

### Participants that Endorsed Birth Trauma

There were 60 participants (41.1%) that endorsed at least one of the DSM-5 criteria for a traumatic event, indicating an experience of birth trauma. Of the 60 participants, participants endorsed one item (*n* = 29, 19.9%), two items (*n* = 13, 8.9%), three items (*n* = 11, 7.5%), and four items (*n* = 7, 4.8%) on the scale. Many participants received a debrief from their providers (*n* = 40, 66.7%), although a sizeable portion did not receive a debrief (*n* = 11, 18.3%). For some participants, it was unclear whether they received a debrief (*n* = 9, 15%; responses included “I don’t remember”, “probably”, or “kind of”). Participants who experienced birth trauma were likely to accept a flyer on a self-guided intervention, Trauma-Focused Expressive Writing. (*n* = 48, 80%) and a handout on psychotherapy services for birth trauma (*n* = 47, 78.3%) when the flyer was offered at the postpartum mother-baby unit.

### Providers Perception of Birth Trauma

Of the 60 participants that endorsed birth trauma, there were 41 providers that also completed the questionnaire while 19 providers (31.6%) did not fill out the questionnaire. Of the 41 completed provider questionnaires, 27 providers (65.9%) also endorsed perceiving the patient’s birth as traumatic while 14 providers (34.1%) did not endorse that the patient’s birth was traumatic. Of the 27 providers who accurately perceived their patient’s birth as traumatic, 18 providers (66.7%) reported debriefing with their patient.

Of the 86 participants that did *not* endorse birth trauma, there were 54 providers that completed the questionnaire while 32 providers (37.2%) did not fill out the questionnaire. Of those who filled it out, 37 providers (68.5%) accurately perceived the birth as not traumatic. However, 17 providers (31.4%) endorsed that their patient experienced birth trauma even though the patient did not themselves report a traumatic birth.

A chi-square test of independence was performed to examine the relation between endorsement of birth trauma by participants and endorsement of birth trauma by providers.

The relation between these variables was significant in the alignment between care provider and patient perceptions of the event, (χ^2^ (1, *N* = 95) = 11.07, *p* <.001). Providers were more likely to accurately perceive their patient’s birth experience the same way the patient perceived it when the patient did not endorse their birth as traumatic.

### Acceptability of Birth Trauma Surveys

Of the participants (*N =* 30) that responded to follow-up qualitative questions, 11 respondents endorsed birth trauma, while 19 did not endorse birth trauma. Tables 3 and 4 illustrate the number of respondents to yes/no questions, main themes, definitions of themes, and example participant quotes.

**Table 3:** Follow-Up Qualitative Survey Responses

Survey Question	Endorsed Birth Trauma		Did Not Endorse Birth Trauma		All Participants	
	Yes *N*(%)	No *N*(%)	Yes *N*(%)	No *N*(%)	Yes *N*(%)	No *N*(%)
Did you want to be asked if your birth experience was distressing or traumatic?	9(81.8)	2(18.2)	10(52.6)	9(47.4)	19(63.3)	11(36.7)
Have you used the tool on how to write a birth story?	0(0)	11(100)	2(10.5)	17(89.5)	2(6.7)	28(93.3)
Have you contacted any IUH resources for follow-up care to address your birth experience?	2(18.2)	9(81.8)	1(5.4)	18(94.7)	3(10.0)	27(90.0)
Did asking about if your birth experience was traumatic affect your experience in the postpartum period?	4(36.4)	7(63.3)	1(5.4)	18(94.7)	5(16.7)	25(83.3)

**Table 4:** Results of Qualitative Analysis

Theme	Definition		Participant Quote	
A. Overall Experience		Participants described their overall birthing experience ranging from their perceptions of doctors and nurses, managing adverse outcomes, to responding to delivery questions.		“It was a great birthing experience despite it being traumatic as I had high blood pressure and developed pulmonary edema. Having someone ask me how it went was helpful in validating how I felt about my experience.”
B. Acceptability Immediately Post-Birth		Participants shared their level of acceptability about being asked to complete the questionnaire about their labor and delivery experience immediately after giving birth.		“I didn’t mind being asked about my experience; however, it was so fresh that I hadn’t really processed any of it or realized the impact it would have on me. I do wish that I would’ve had more of an opportunity to debrief about what happened during my labor/delivery. I’m 7 months postpartum now and still don’t fully know what happened or what implications my complications have for the future.”
C. Ways to Increase Comfort		Participants indicated if there was anything that could have been done to make them feel more comfortable, as labor and delivery can be distressing or traumatic for some people.		“I didn’t find it uncomfortable. The only thing I can think of would be to give a little more information about who you are and the purpose of this study. It may also be helpful to ask the same questions again on a follow-up survey after parents have had time to process things a bit.”
D. Acceptability of Length		Participants commented on the length of the 5–10-minute survey and how long it took them to complete the questionnaire.		“I would say keeping it to 5 min will get more birthing people to complete it since the healing and taking care of a newborn is very taxing.”
E. Most Helpful		Participants noted what was most helpful about being asked about their labor and delivery experience.		“I think that asking if a patient would like follow-up is helpful. Although, it’s really only helpful if there is an intention to help make that happen. I wouldn’t mind really talking things through with the people who were there. I’ve gotten bits and pieces from my husband and some of the people who cared for me but I’m left piecing together my ‘birth story’. I feel like processing things with the people who were actually there and know what happened has been more beneficial than any amount of therapy.”
F. Not Helpful	Participants identified areas or information they disliked being asked about after childbirth.		“Not really, I hesitated to directly share negative experiences of the doctors, but I tried to be fair while also sharing what happened. The positive experiences did outweigh the negative.”	
G. Postpartum Healing	Participants discussed how being questioned about their birth experiences, including whether they were traumatic, influenced their postpartum recovery.		“That acknowledgment allowed me to fully accept that my birth experience was difficult and warranted time and resources to process.”	

###  A. Overall Experiences

Within this theme, participants depicted responses as *positive* “Good,” *negative* “Could be improved,” or *neutral* “Birth was traumatic, doesn’t blame team” reactions to their birth experiences. Most participants (*n* = 19, 63.3%) endorsed that they wanted to be asked whether their birth experience was distressing or traumatic, especially those that had experienced birth trauma (*n =* 9/11 (81.8%))

####  B. Acceptability Immediately Post-Birth 

Most participants (*n* = 24, 80%) indicated completing the survey after birth was acceptable or beneficial to them. For example, one participant noted that completing it was “great, I got to process my experience.” Very few participants described completing the survey as difficult or had specific feedback about the timing.

####  C. Ways to Increase Comfort

Most participants (*n* = 23, 76.6%) indicated that there was nothing that could have been done to make them feel more comfortable.

####  D. Acceptability of Length

The survey took about 5–10 min to complete, and participants were asked to comment on the length of the survey. Most participants agreed that the survey was a “fine” or appropriate length (*n* = 23, 76.6%), as anything longer could generate less engagement which is evident in this participant’s comment, “It was fine, any longer would be overwhelming.”

####  E. Most Helpful

Participants identified the types of information they found most helpful to be asked about and appreciated having the opportunity to share their experiences. Some thought it was beneficial, such as this respondent, “Being able to talk about my experience immediately afterward was therapeutic.” Others deemed it necessary like this participant, “It was good to be asked directly if I found my birth traumatic. I tend to not want to be dramatic, but I had a 38-hour labor that ended in a C-section, so there was a lot of delayed emotion that needed to be processed after physical/emotional safety was re-established.” Participants also noted it was helpful being asked how improvements could be made to the birthing experience and that they generally appreciated that the team cared and that their responses could help other mothers.

####  F. Not Helpful

None of the participants identified anything that they disliked being asked during their experiences.

####  G. Postpartum Healing

Most participants (*n* = 25, 83.3%) indicated that being asked about if their birth experiences were traumatic did not affect their postpartum recovery. Those who indicated that it had affected them in the postpartum period only reported positive effects (“I feel like talking about it helped with moving forward”; “That acknowledgment allowed me to fully accept that my birth experience was difficult and warranted time and resources to process”).

Most participants (*n* = 28, 93.3%) did not use the writing a birth story tool that was provided. In addition, very few (*n* = 3, 10.0%) participants used the IUH resources that addressed follow-up care on birth experiences. Thus, participants appeared to benefit from being asked about their birth experiences, valued the opportunity to process these experiences—particularly when they were distressing or traumatic—and considered such conversations to be clinically appropriate when approached with empathy, respect, and sensitivity.

## Discussion

This study sought to understand how often providers and patients perceived the same birth experience as traumatic and subsequent communication patterns. While there were higher rates of birth trauma (40%) than previously found in the literature, only two out of three providers accurately perceived their patient’s birth to be traumatic (Heyne et al. 2022). Further, about one in three providers perceived their patient’s birth to be traumatic when the patient did not perceive it that way. This is somewhat inconsistent with the literature that demonstrates that providers and those who describe their birth as traumatic tend to describe the same types of events as traumatic (e.g., stillbirth/infant death, instrument injury to participant or newborn during the birth process; Alcorn et al. 2010; Robinson et al., 2022), highlighting the role that perception of the event, emotional response, and lack of understanding can have on birth trauma.

For communication patterns, only 67% of participants that experienced birth trauma definitively had conversations with their providers post-delivery. It could be possible that positive perception of their birth could influence a provider’s willingness to debrief with their patient, or that providers who may have perceived their patient’s trauma but then lacked confidence or comfortability to debrief with them. Lastly, nearly all participants found being asked about birth trauma neutral to acceptable, with some finding the experience of being asked validating and helpful, especially if they experienced birth trauma.

When considering the communication patterns we found, our findings are consistent with the literature that describes a hesitance of providers, rather than their patients, in talking about trauma exposure (Green et al. 2011; Goldstein et al. 2017). For example, Goldstein et al. (2017) found that 4 out of 5 primary care patients endorsed this statement being asked about adverse child experiences (ACEs), “I am comfortable being asked about ACEs by my clinician”, regardless of a participant’s number of ACEs. Further, providers have reported that it is critical to have an established workflow for how to help their patients (e.g., referrals to psychiatry, social work) if trauma exposure/PTSD symptoms are disclosed (Green et al. 2011; Flanagan et al., 2018). It is important to ensure that obstetric providers know that trauma-informed care for those who have experienced birth trauma is separate from trauma-focused psychotherapy. This is similar to the effect that providing education and communicating with patients can improve surgical outcomes. For example, less pain medication is needed and there are decreased episodes of hypotension in the operating room when preoperative anxiety is lowered when education is provided (Brodersen et al. 2023; Khorfan et al. 2020; Orbach-Zinger et al. 2012).

One solution is to create a universal workflow for brief conversations with the patient post-delivery asking about the birth experience, including if they found it extremely stressful or traumatic, and if so, helping patients to understand what happened during delivery as well as providing basic validation and support. This routine conversation should not take much time or require advanced mental health knowledge; this could also be an ideal time to pull in other team members to offer more support and resources if needed (e.g. nursing staff, patient liaison, social worker). It is also important to clarify that debriefing after birth trauma is not the same as psychological debriefing, which is a lengthy, often group-based psychological intervention used following a traumatic event to prevent PTSD that has not been found to be effective (Foa et al. 1995; McNally et al. 2003; Wessely et al. 2002).

Participants did not find being asked about birth trauma particularly distressing and for those who experienced birth trauma, being asked was often validating and meaningful. This is consistent with seminal studies that have disproven that asking or talking about trauma worsens psychological symptoms (Becker-Blease & Frey, 2006; Edwards et al. 2009). Most participants do not find being asked about trauma histories is distressing nor do they feel re-traumatized (Reynolds et al., 2026). Not only does directly assessing abuse histories and related symptoms not cause harm, but individuals may view it as positive or report lower distress because of being asked about these topics (Hemenover 2003; Kirkner et al. 2019; Reynolds et al., 2026).

Additionally, about a third of our sample identified as Black/Latina. Racially and ethnically minoritized women are more likely to experience disproportionate rates of obstetric discrimination or trauma, increased communication barriers, unconscious/implicit bias, pain management disparities, and their concerns being minimized or dismissed (Iyengar et al. 2022; Johnson et al., 2019). Future research needs to explore if the discrepancies between experiences is an indicator of more systemic bias playing a role in the birthing experience of the patient.

### Limitations

There are some significant limitations to this study, the biggest of which is missing data from providers. There were no incentives to complete the survey, and providers already have several demands on their time immediately post-delivery. Additionally, we did not have data on providers’ previous training or frequency of birth traumas they have encountered, which could have influenced how they evaluated if a patient experienced their birth as traumatic. Other studies have used a provider-specific measure to assess trauma exposure, including how frequently a provider observed several potentially traumatic obstetric events (e.g., maternal death, stillborn birth), how severe they considered each traumatic event, and what effect witnessing a patient’s birth trauma had on their personal and/or professional life (Robinson et al., 2022). Future research would benefit from a more systematic assessment of (1) birth trauma, (2) communication patterns with providers, (3) how provider backgrounds and training affect their perception of patient trauma, including with a provider-specific measure to assess trauma exposure. Our team is working to standardize a birth debrief checklist to increase the chances of a post-delivery conversation routinely happening, yet more research is needed from both the providers’ and participants’ perspective. An additional limitation is that we only talked with individuals who had delivered a living child. Further, the study was only conducted in one hospital unit and there may have been bias in who chose to participate or not. We may be missing data from those who had very negative or positive birth experiences, who didn’t want to discuss their experience because it was too difficult or because they viewed it very positively. Lastly, we do not have data on the effects associated with our sample who experienced birth trauma. More information is needed to understand how the experience or lack of birth trauma/provider debriefs affects long-term maternal and infant functioning.

## Data Availability

The data that support the findings of this study are available on request from the corresponding author. The data are not publicly available due to privacy or ethical restrictions.

## Declarations

### Clinical Trial Number

N/A.
